# Overview of the CMS Innovation Center’s Strategic Vision and Development of the GUIDE Model

**DOI:** 10.1002/alz.095810

**Published:** 2025-01-09

**Authors:** Purva Rawal

**Affiliations:** ^1^ CMS Innovation Center at Centers for Medicare & Medicaid Services, Washington, DC USA

## Abstract

N/A N/A N/A N/A N/A N/A N/A N/A

